# Editorial Notes

**Published:** 1939

**Authors:** 


### Editorial Notes

**Dr. A. L. Flemming.**

When Dr. A. L. Flemming became President of the Bristol Medico-Chirurgical Society in 1926 an Editorial Note mentioned that he had consented to continue as Editorial Secretary to the Journal Committee, because his experience as Manager was indispensable at a time when the Editorial Committee had undergone so many changes. This was on the occasion of the resignation of the late Dr. P. Watson-Williams from the Editor's Chair. During the subsequent thirteen years Dr. Flemming has continued to act as Secretary to the Editorial Committee. In spite of ill-health his diligence in looking after the business side of the *Journal* has never relaxed. His attendance at meetings was always regular, and it was amazing to see him come cheerful and full of sound ideas when his sufferings were intense. The Society and the *Journal* owe much to Arthur Flemming's loyal support, and his colleagues in the Editorial Committee desire to record their gratitude for his help and guidance.

**The War.**

Once more the shadow of war has come upon us. Not with the dramatic unexpectedness of 1914, but in spite of widespread hopes that the problem of Poland might be settled peacefully. Thus the country was not unprepared and when war was declared on

3rd September it caused little excitement; there were no outbursts of Jingoism, the feeling most generally expressed was one of annoyance. In comparison with 1914 the medical profession in Bristol and the West has been remarkably undisturbed. It is true that as a precaution our general hospitals were rapidly emptied by an order that was dictated perhaps by panic, perhaps by prudence, but certainly not by common sense. Some unnecessary suffering was inflicted on the patients so unceremoniously bundled home, and some hardship created for those who were in urgent need of hospital treatment because the hospitals could not admit them. However, the policy of keeping vast numbers of beds empty for civilian casualties was soon abandoned and a more reasonable view now prevails. From the beginning the hospital staffs have never been withdrawn from their ordinary duties and under the Group Officer, Professor Hey Groves, numerous "teams" have been formed so that, if the necessity arises, the re-arranged staffs of our hospitals will be capable of dealing with casualties either civil or military in the manner they were dealt with in the Expeditionary Force in France by the end of the last war. This has been achieved with the minimum of disturbance and with infinitesimally less expense than was incurred in 1914. Then civil hospitals were most inconveniently renamed Territorial General Hospitals, and their staffs given military uniforms and military titles. The maximum interference was devised for preventing the normal work of staffs being carried on in the normal way. The duties of surgeons and physicians to their patients are the same whether the patients belong to the fighting services or to the civil community, and in the present war this fact looks like being appreciated.

Of the Air Raid Precautions it is wiser to say no more than that so far their efficiency has luckily not been put to the test. If the dangers from the air have been over-estimated it cannot be a matter for regret that the country was fully prepared for something worse than the reality. It is far too soon to speak. We must be thankful if our preparations never are put to the test. The organization of the medical profession under the Central Emergency Committee and the various local Emergency Committees has up to the present worked fairly well, but it must be remembered that the requirements of the fighting services for medical personnel have as yet been very small. When heavier demands begin to be made the machinery that has been devised will be put to much severer tests. The University has begun to feel something of the pressure. An influx of students from London University into Bristol has taken place and we are accommodating a large number of students and teachers from King's College and pre-medical students from the Middlesex Hospital. They are welcome guests and we hope that they are finding the arrangements for their accommodation adequate and comfortable. The "blood-transfusion" centre established at Southmead Hospital is arousing great interest and is splendidly staffed. Bristol is fortunate in being classed as a "neutral area." One new departure is of great importance: the Final Examinations of the Conjoint Board have been partly decentralized from London and four provincial centres have been selected in which the examinations have been held for the first time. Of these provincial centres Bristol, on account of its geographical situation and its excellent clinical facilities, is one.

**The 144th Field Ambulance.**

ONE R.A.M.C. unit has been raised in Bristol, the 144th Field Ambulance (Territorial Army). It is commanded by Lieut.-Colonel W. A. Jackman, T.D., and was embodied on declaration of war. This unit is the successor to the 3rd South Midland Field Ambulance, which went overseas with the 48th Division from 1915–1918 and saw much service in France and Italy. The 144th Field Ambulance forms part of the present 48th Division: its personnel is as follows:—

Lieut.-Colonel W. A. Jackman, T.D., M.B., F.R.C.S., R.A.M.C.
Major E. W. Ashworth, M.B., R.A.M.C.
Capt. J. D. A. Gray, M.B., F.R.C.P.E., R.A.M.C.
Lieut.-Q.M. T. E. Holloway, R.A.M.C.

| | |
|---|---|
| Capt. V. H. Garland.<br>Capt. R. G. W. Whitfield.<br>Lieut. A. G. Walter.<br>Lieut. G. Forrest Hay.<br>Lieut. M. M. Lewis.<br>Lieut. D. K. Moynagh.<br>Lieut. R. J. K. Tallack.<br>Lieut. R. K. Pilcher. | R.A.M.C. |

*Attached.*

Lieut. C. R. H. Fenton, Army Dental Corps.
Lieut. J. O. Penrose, R.A.S.C.

165 Other ranks R.A.M.C.
56 Other ranks R.A.S.C.

221+14 Officers at present.

Colonel Jackman writes from one of England's Stately Homes where "we are, at present, running a small hospital of twenty beds for people with colds and training hard in field work." "I personally," he says, "haven't seen a case or touched an instrument (other than musical) since we were mobilized. I have seen Miss Johnston (formerly Matron of the B.R.I.), and had a chat with her ; she is going to get a job in the Army Nursing Service in the fulness of time. Jenkins and Hooper, who joined up with us, have gone to other units. I am absolutely overwhelmed with correspondence, mostly official, but I am always pleased to hear Bristol news: let me know how things are going if you can ever spare a moment."

### Obituary

